# A Comparison of Capsular Contracture Rates after Implant-Based Breast Reconstruction Using ADM Versus Synthetic Mesh

**DOI:** 10.1093/asjof/ojae007.018

**Published:** 2024-04-12

**Authors:** Jennifer Bai, Sarah Ferenz, Megan Fracol, John Y S Kim

**Affiliations:** Northwestern Memorial Hospital, Chicago, IL; Northwestern Memorial Hospital, Chicago, IL; Northwestern University Feinberg School of Medicine, Chicago; Northwestern Memorial Hospital, Chicago, IL

## Abstract

**Goals/Purpose:**

Capsular contracture is a local complication in which there is tightening of the capsule around the implant. This complication has been shown to occur in 15-30% of implant-based breast reconstruction patients and often requires revision surgery for correction. Prior investigations have suggested that the use of ADM can reduce the risk for development of capsular contracture, with more recent research suggesting that synthetic mesh may provide the same benefit. Several meta-analyses have attempted to compare ADM and mesh use for the prevention of capsular contracture, but they all emphasize the continued need for clinical investigation with direct comparisons. The goal of this study is to assess our own reconstruction patient population to compare capsular contracture rates between ADM and mesh cohorts, as well as to assess for other potential risks that may increase the rate of capsular contracture in these patients.

**Methods/Technique:**

A 15-year retrospective review of all implant-based breast reconstructions performed by the senior author was performed. All operations occurred between 2008 and 2023 in a hospital setting. Patient demographics were assessed including age, BMI, implant size, radiation history, location of malposition, and use of either ADM or mesh. Post-operative complications including incidence of capsular contracture were recorded. Patients who had documented capsular contracture prior to scaffold insertion were excluded from the study. Wilcoxon signed rank test, 2-sided t-test, and Fischer’s Exact test were used to compare baseline demographics and capsular contracture rates between the two groups, along with a multivariate logistic regression analysis to control for potential confounders.

**Results/Complications:**

Fifty-two breasts underwent capsulorraphy, of which 25 (48.1%) used ADM and 27 (51.9%) used mesh. Average age was 50.6 years, average BMI was 27.7, average implant size was 541.8 cc, and eight breasts (15.4%) had been irradiated. Average follow-up time was seven years (mean = 85.4 months, range 22 – 149 months, SD = 35.9). Fourteen (26.9%) capsulorraphies were for inferior malposition, 13 (25.0%) for lateral malposition, and 25 (48.1%) were for inferolateral malposition. Patients in the ADM group had significantly more lateral malposition prior to insertion as compared to the mesh group which had more inferior malposition (p=0.0246). There was no significant difference between the two groups for any other recorded baseline demographic; these demographics included age, BMI, hypertension, diabetes, history of tobacco use, radiation, chemotherapy, implant volume, and implant surface texture. Capsular contracture occurred in 2 ADM breasts (8.0%) and 4 mesh breasts (14.8%) with no significant difference between the two groups (p=0.6695). Five of these capsular contractures had documented Baker grades with three grade II, one grade III, and one grade IV. None of the recorded baseline demographics significantly increased the risk for capsular contracture development.

**Conclusion:**

ADM and synthetic mesh both function as effective tools for reducing the risk of developing capsular contracture after prosthetic breast reconstruction. The rate of capsular contracture with ADM was only 8.0% and the rate with mesh was only 14.8%. Both rates are lower than the 15-30% incidence that has been found in breast reconstruction patients without mesh. None of the patient demographics were significant risk factors for capsular contracture development, so these are unlikely to be confounders. There was no significant difference in rate of capsular contracture between the two groups, suggesting that ADM and mesh are similarly efficacious at preventing capsular contracture in reconstruction patients.

